# Fair surface modification with mixed alkanethiols on gold nanoparticles through minimal unfair ligand exchange†

**DOI:** 10.1039/d4na00270a

**Published:** 2024-07-16

**Authors:** Kun Xiong, Masaharu Nagayama, Kuniharu Ijiro, Hideyuki Mitomo

**Affiliations:** a Graduate School of Life Science, Hokkaido University Sapporo 060-0810 Japan; b Research Institute for Electronic Science, Hokkaido University Sapporo 001-0021 Japan mitomo@es.hokudai.ac.jp

## Abstract

Surface modification with functional molecules is essential for introducing various surface properties. As gold nanoparticles (AuNPs) have extraordinary chemical, physical, and optical properties, control of their surface, mainly through modification with mixed alkanethiols *via* Au–S interactions, has attracted much attention. However, surface modification of AuNPs with mixed alkanethiols to provide a strictly regulated composition remains challenging. Further, there are very few methods that can easily establish the nature of ligands and their replacement with similar molecules at nanoparticle surfaces, limiting precise analyses. Herein, we demonstrate an unfair ligand exchange between oligo(ethylene glycol) (OEG)-attached alkanethiols as a source of unfair surface modification utilizing programable thermo-responsive properties of OEG-alkanethiols-modified AuNPs and fair surface modification with mixed OEG-alkanethiols by minimizing this effect. OEG-alkanethiols-modified AuNPs show an assembly/disassembly behavior in response to the solution temperature. Assembly temperature (*T*_A_) changes in the presence of other OEG-alkanethiols, confirming the ligand exchange between alkanethiols in an aqueous solution. Kinetic analyses indicate that the competitive exchange reaction of these two alkanethiols results in an unfair ligand exchange, which leads to gradual changes in surface composition. As this ligand exchange between alkanethiols takes a longer time compared to that from citric acid, which initially covered the AuNPs, exact surface modification of AuNPs with OEG-alkanethiols is performed by moderate reaction conditions (25 °C, several to 24 hours). This insight regarding “more prolonged reaction is not always better” could be widely applied for surface modifications with various thiol-ligands.

## Abstract 2

Ligand exchanges between alkanethiols at gold surfaces were demonstrated as a cause of unfair surface modification of gold nanoparticles with mixed alkanethiols. Kinetic analyses suggested an importance of moderate reaction conditions.

## Graphical abstract

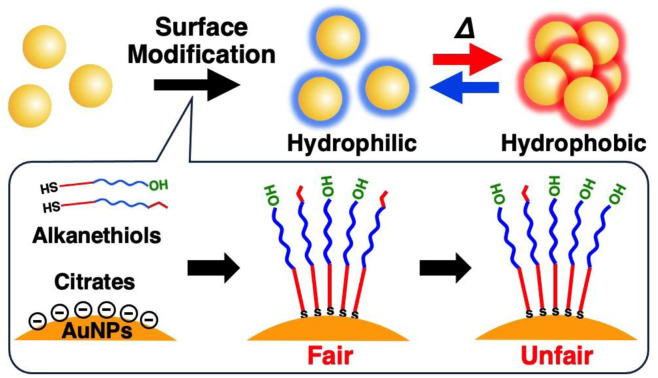

## Introduction

Surface modification with functional ligands plays an essential role in providing nanoparticles with desirable properties such as good dispersibility^1–3^ and stimuli-responsiveness.^4–8^ As gold nanoparticles (AuNPs) show plasmonic absorption^9–11^ and interparticle plasmon coupling on assembly,^12–14^ the stimuli-responsive, particularly thermo-responsive, assembly of AuNPs is anticipated for a wide range of applications.^15–18^

To date, synthetic polymers are widely used as stimuli-responsive ligands. Poly(*N*-isopropyl acrylamide) (pNIPAM) is well known as a thermo-responsive polymer showing phase transition through hydration/dehydration over lower critical solution temperatures (LCSTs).^19–21^ Even though synthetic techniques for polymers have been well-developed, these polymers possess some heterogeneity in molecular sizes and present with various conformations, leading to a broad range of stimuli-responsiveness on AuNP surfaces. Their large sizes are also thought to result in large interparticle gaps on AuNP assemblies, resulting in a weak plasmon coupling effect.^22^

On the contrary, small molecular ligands present with equivalent conformations based on their definite chemical structure with uniform molecular size, allowing sharp stimuli-responsiveness. A thin surface coating of AuNPs with small molecules is also supposed to enhance the plasmon coupling effects. Alkanethiols are small ligands and are one of the most widely used molecules for surface modification. They exchange ligands with the capping molecules without thiol groups, such as citrate or cetyltrimethylammonium bromide (CTAB), on the AuNP surface due to robust Au–S bonds,^23–25^ and then self-assemble into highly packed, homogeneous, and stable monolayers, known as self-assembled monolayers (SAMs) with the aid of alkyl chains.^26–28^ Surface modification with mixed alkanethiols affords an easy but reliable way to provide various tunable surface properties.^29,30^ For example, the mixture of alkanethiol molecules with carboxylate and trimethylammonium groups has been applied to precisely tune pH-responsiveness on AuNP assembly to the tumor acidic microenvironment for cancer therapy.^31–33^ Also, the mixture of oligo(ethylene glycol) (OEG)-attached alkanethiol molecules with hydroxyl and carboxylate groups has been applied to control protein adsorption on AuNP surfaces.^34^ Nevertheless, it remains challenging to drive mixed ligand-immobilization on the AuNP surface in proportion to the mixing ratios in solution as fair surface modification (or fair ligand exchange). In other words, such reactions often induce unfair, or uneven, surface modification, resulting in disproportionate ratios of mixed ligands at the surface against those in solution.^35,36^ Many researchers attribute this unfairness to differences in the properties of the ligands, such as charge, polarity, solubility, and bulkiness, in the ligand exchange from citrate or CTAB to alkanethiols.^37–39^ On the other hand, it has been reported that alkanethiols (thiolates) immobilized on the Au surface can be replaced with free alkanethiols in organic solvent.^40–47^ It is expected that this ligand exchange between alkanethiols also causes unfair surface composition during modification. Despite their broad applications, no reports provide a clear answer regarding fair surface modification with mixed ligands from the perspective of alkanethiol ligand exchange probably due to the difficulty in the analyses. There are very few methods that can easily establish the nature of ligands and their replacement with similar molecules at nanoparticle surfaces.

**Scheme 1:**
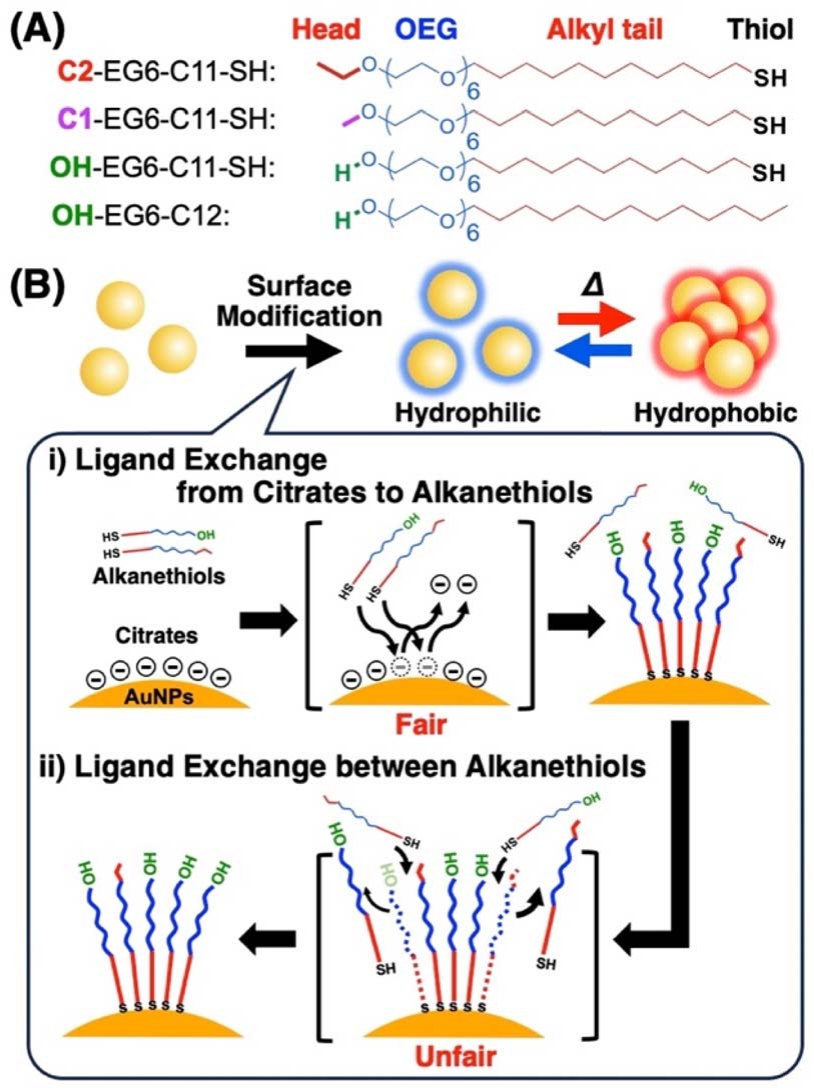
(A) Chemical structures of the OEG-alkanethiol derivatives used in this study and (B) an illustration of the surface modification of AuNPs with mixed alkanethiol ligands, providing thermo-responsive properties. Surface modification includes (i) ligand exchange from citrates to alkanethiols as an early quick reaction and (ii) ligand exchange between alkanethiols as a late slow reaction. Surface composition was determined from assembly temperature on heating.

We have previously reported that surface modification with OEG-attached alkanethiol ligands, which are biocompatible molecules, provided thermo-responsive properties to AuNPs, that is, they could assemble/disassemble in response to temperature in an aqueous solution through changes in the surface property, hydrophilic/hydrophobic, *via* the hydration/dehydration of the OEG portion, like pNIPAM.^48,49^ Further, precise tuning of this thermo-responsive property has been programmed *via* mixing two kinds of OEG-alkanethiols with different head groups.^50^ However, the ligand composition on the surface seems not to completely match the mixing ratio of each ligand, even though the ligands used have similar chemical structures, which could provide an equivalent attachment rate constant. To clarify this point, in this study, we focused on the ligand exchange between free OEG-alkanethiols and OEG-alkanethiols (thiolates) attached to the Au surface, and demonstrated unfair ligand exchange between OEG-alkanethiols after the ligand exchange from citric acid by utilizing programable thermo-responsive properties of OEG-alkanethiols-modified AuNPs (Scheme 1). Finally, we prepared thermo-responsive AuNPs with an exact surface composition of the mixed ligands *via* minimizing ligand exchange between OEG-alkanethiols as a source of unfairness.

## Results and discussion

### Thermo-responsive AuNPs modified with OEG-alkanethiols

**Fig. 1:**
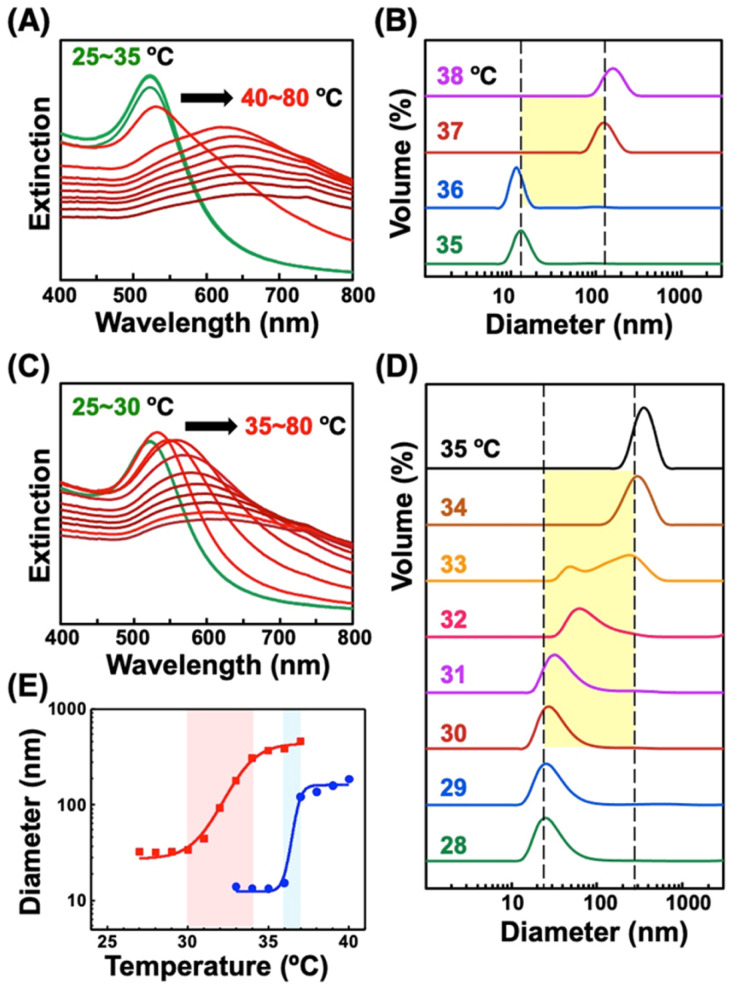
Thermo-responsive phenomena of AuNPs modified with (A, B) C2-EG6-C11-SH and (C, D) pNIPAM-SH upon heating. (A, C) Extinction spectra and (B, D) size distribution determined by DLS. (E) Size changes of AuNPs@C2-EG6-C11-SH (blue) and AuNPs@pNIPAM-SH (red) upon heating. Thermo-responsive properties of AuNPs@pNIPAM-SH were measured in 20 mM NaCl aq. Black arrows in (A) and (C) indicate plasmon peak shifts.

First, we prepared AuNPs modified with a single kind of OEG-alkanethiol, C2-, C1-, or OH-EG6-C11-SH. The AuNP (diameter of 10 nm) surfaces were modified with OEG-alkanethiols *via* ligand exchange reaction in water according to the method described in our previous paper.^50^ Extinction spectra, size distribution, and zeta-potential changes support successful surface modification (Fig. S1 and Table S1†).^48–50^ Then, we confirmed their thermo-responsive surface property changes by extinction spectral and DLS analyses, as these cause the assembly/disassembly of AuNPs. Large spectral changes (a peak shift of more than 150 nm) over 35 °C supported the interparticle plasmon coupling among 10 nm AuNPs@C2-EG6-C11-SH, indicating the thermo-responsive assembly of AuNPs occurred (Fig. 1A, S2-blue). DLS measurements showed that their size changed from *ca.* 10 nm to *ca.* 100 nm abruptly between 36 and 37 °C (within 1 °C), indicating their assembly formation *via* the surface hydrophobicity change with sharp thermo-responsiveness (Fig. 1B, 1E-blue). The assembly temperature (*T*_A_) was determined from DLS as 36.5 °C, as DLS simply provides assembly information, while plasmon shifts include complex information. Similarly, the *T*_A_ of 10 nm AuNPs@C1-EG6-C11-SH was determined to be 76.5 °C (Fig. S3A†). In the case of AuNPs@OH-EG6-C11-SH, no AuNP assembly was observed within 80 °C, indicating their *T*_A_ is over 80 °C (Fig. S3B†).^48^ Here, we compared thermo-responsive properties of our OEG-alkanethiol-modified AuNPs to those of conventional polymer (thiol-terminated pNIPAM; MW = 6000)-modified AuNPs. The 10 nm AuNPs@pNIPAM showed a smaller red-shift (*ca.* 80 nm) on assembly formation by spectral analyses, indicating polymers provide larger gap distances on assembly due to their larger molecular sizes (Fig. 1C, S2-red). DLS results showed their size was *ca.* 30 nm at the lower temperature as dispersed state due to their larger surface ligands and changed to *ca.* 300 nm (as assembled) between 30 and 34 °C, indicating their *T*_A_ was *ca.* 32 °C across a broader response range of *ca.* 4 °C, even though their polymer chain length was relatively low (Fig. 1D, 1E-red). It has been reported the oligo-NIPAM solution shows thermo-responsive properties (clouding temperature) at a high concentration, and a higher concentration provides a sharper responsiveness,^19^ supporting the notion that the use of small molecules at high density is a better approach than that of polymers. These results indicate our OEG-alkanethiol-based AuNPs have better potential.

### Ligand exchange between alkanethiols on AuNPs

**Fig. 2:**
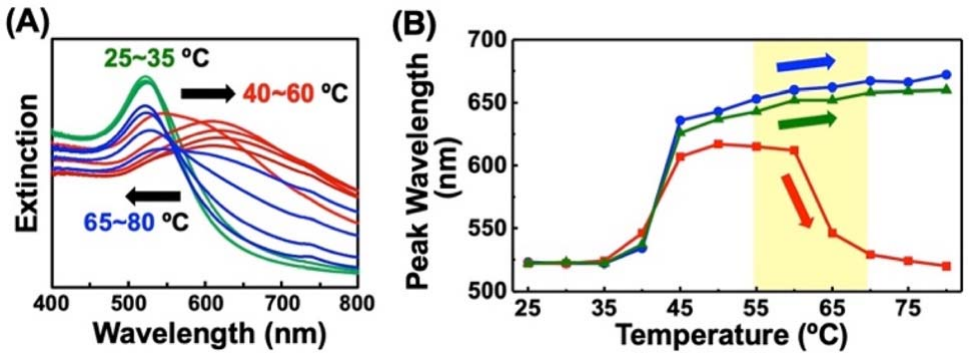
Extinction spectra of AuNPs@C2-EG6-C11-SH upon heating in the presence of OH-EG6-C11-SH. (B) Extinction peak shifts at each temperature of AuNPs@C2-EG6-C11-SH in the absence of free ligands (blue), in the presence of free OH-EG6-C11-SH (red), and in the presence of OH-EG6-C12 as an OEG-alkanethiol derivative (green). Arrows in (A) and (B) indicate plasmon peak shifts.

To investigate ligand exchange between alkanethiols on AuNP surfaces (Scheme 1B(ii)), AuNPs@C2-EG6-C11-SH was mixed with free alkanethiol derivatives. AuNPs showed unique spectral changes in the presence of free OH-EG6-C11-SH ligands (Fig. 2A). The peak shift profile upon heating is shown in Fig. 2B. Although AuNP@C2-EG6-C11-SH, as original particles, only showed a red-shift over 35 °C (Fig. 2B-blue), in the presence of the external OH-EG6-C11-SH ligands, they show not only a red-shift over 35 °C, which is same as the result as for the original particles, but also a blue-shift over 60 °C, indicating their return to a dispersed state upon heating (Fig. 2B-red). To investigate this phenomenon, these AuNPs, after the measurement, were purified by centrifugation to remove free ligands in the solution and then analyzed. These AuNPs showed no spectral shift on heating up to 80 °C (Fig. S4A†). DLS analysis also supported that AuNPs didn't assemble in this temperature range (Fig. S4B†). The above findings indicated that the thermo-responsiveness had been changed with the aid of the free alkanethiol ligands. On the contrary, in the presence of OH-EG6-C12 molecules, which have a similar chemical structure to the OH-EG6-C11-SH ligand but no thiol group, the spectra and DLS measurement showed unchanged thermo-responsiveness (Fig. 2B-green, S4C, and D†). These results suggested that the free alkanethiols of OH-EG6-C11-SH replaced the immobilized C2-EG6-C11-SH on the AuNP surface, as our previous research showed the *T*_A_ significantly increases on increases in the surface composition of OH-EG6-C11-SH.^50^ In the same way, the addition of C1-EG6-C11-SH to AuNPs@C2-EG6-C11-SH also caused changes in thermo-responsive properties (Fig. S5†). The *T*_A_ of 51.5 °C is higher than that of the original particles of AuNPs@C2-EG6-C11-SH (36.5 °C), but lower than that of AuNPs@C1-EG6-C11-SH (76.5 °C), suggesting that the free alkanethiol ligands of C1-EG6-C11-SH partially replaced the immobilized C2-EG6-C11-SH.

To further confirm the ligand exchange between the alkanethiol ligands, we added free C2-EG6-C11-SH into the above samples (Fig. S6†). The non-thermo-responsive AuNPs shown in Fig. S4A and B† were purified and then incubated with free C2-EG6-C11-SH at 85 °C for 30 minutes. After purification, DLS measurement showed that the *T*_A_ had fallen from immeasurable value (>90 °C) to 40.5 °C, which is close to the original *T*_A_. This indicates that the free C2-EG6-C11-SH replaced the immobilized OH-EG6-C11-SH and the surface composition returned to the original. This result strongly supports the idea that the ligand exchange between alkanethiols is reversible.

It is well-known that alkanethiol SAMs on a flat gold film are stable.^26–28^ Here, we confirmed the stability of OEG-alkanethiol SAMs on the curved surfaces of AuNPs to investigate the relationship between their stability and ligand exchange. The AuNPs@C2-EG6-C11-SH was heated from 25 to 85 °C followed by cooling from 85 to 25 °C in four cycles during spectroscopic measurement. The spectra and DLS analyses showed no change in their responsive temperature (*T*_A_) in response to four heating cycles (Fig. S7†). As our previous study showed that *T*_A_ depends on the local density of the OEG-portion in alkanethiol ligands,^50^ this unchanged *T*_A_ indicates the unchanged local OEG density at the AuNP surface without detachment, supporting the excellent stability of OEG-alkanethiol SAMs. These findings indicated that free alkanethiols could replace alkanethiols in highly packed SAMs on AuNP surfaces even though they are stably immobilized, suggesting molecular replacement like that of a Newton's cradle.

### Kinetic aspects of ligand exchange between alkanethiols

**Fig. 3:**
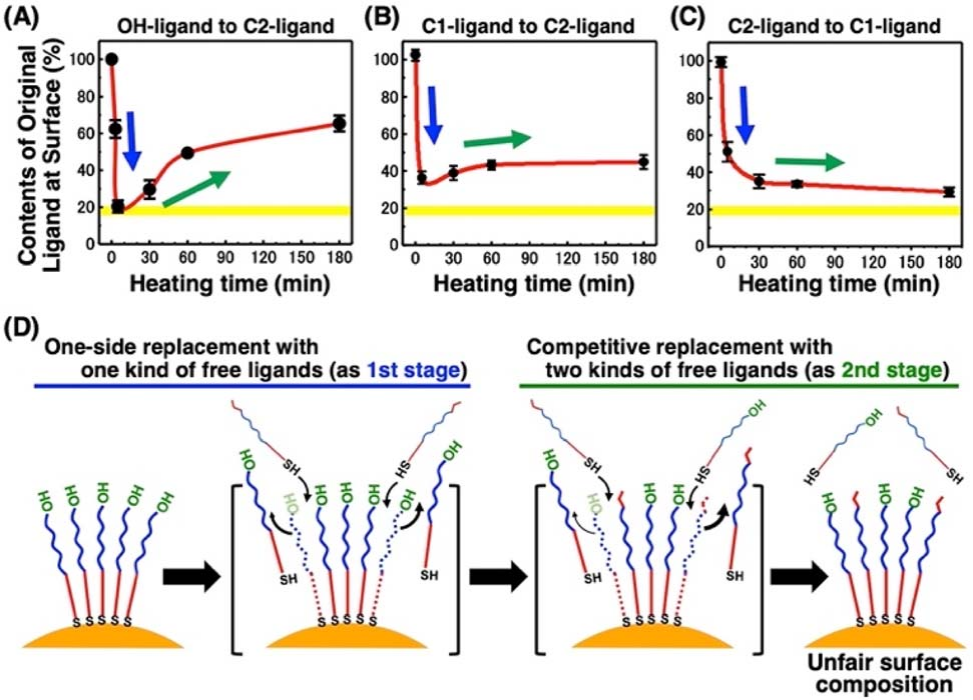
Dynamic changes in the content of original ligands at the AuNP surface for the time-course experiment at 85 °C of (A) 10 nm AuNPs@OH-EG6C11-SH in the presence of C2-EG6-C11-SH, (B) 10 nm AuNPs@C1-EG6-C11-SH in the presence of C2-EG6-C11-SH, and (C) 10 nm AuNPs@C2-EG6-C11-SH in the presence of C1-EG6-C11-SH. (D) Schematic illustration of this ligand exchange reaction. Yellow highlights in (A), (B), and (C) indicate the expected ligand content for fair ligand exchange as the molar ratio of the ligands in the system. Error bars represent SD (*n* = 3).

To obtain detailed information on the alkanethiol exchange reaction, time course experiments were performed. For instance, we added free C2-EG6-C11-SH ligands to the AuNPs@OH-EG6-C11-SH and then incubated them at various temperatures. After various incubation times, AuNPs were quickly cooled down and then purified at 4 °C, followed by DLS measurement. The *T*_A_ dramatically decreased to *ca.* 50 °C after 5 minutes of incubation at 85 °C in the first stage, and then, interestingly, the *T*_A_ began to increase with time during the second stage (Fig. S8†). These *T*_A_ changes are translated to the ligand contents at the AuNP surface based on Fig. 5B, shown later as a linear calibration curve, and the results are plotted as the original ligand content at the surface (Fig. 3A). In the first stage, C2-EG6-C11-SH dominantly replaces the immobilized OH-EG6-C11-SH ligands due to the presence of only free ligands in the external solution, causing the considerable reduction in *T*_A_ (*i.e.*, replacement with C2-EG6-C11-SH) within a short time (Fig. 3A and 3D-blue). It is worth noting that the ligand content of *ca.* 20% at 5 min is close to the calculated ligand molar ratio in the system, originally 15–18 μM of OH-EG6-C11-SH at the AuNP surfaces and 75 μM of C2-EG6-C11-SH in solution as free ligands. In the second stage, ligand contents at the AuNP surface change in an inverse manner, indicating the ligand exchange from C2-EG6-C11-SH to OH-EG6-C11-SH predominantly occurred under competition between OH-EG6-C11-SH and C2-EG6-C11-SH (Fig. 3A and 3D-green). This ligand exchange reaction in the second stage leads to a deviation from *ca.* 20% of evenly modified ligand content at the AuNP surfaces to an uneven ligand content of over 60%. For the binding sites of alkane-thiols, it is known that AuNPs possess several facets, such as (111) and (100), with varied stability of thiolates on them.^51^ This may provide any effect on the ligand exchange reaction. However, our results show that the ligand exchange ratio at the surface is large and heading to the ligand ratio in the solution (Fig. 3), suggesting whole ligand molecules are replaceable, even though there might be some difference depending on the facets. This corresponds to the claim that the NP surface properties approximate those of SAMs on planar surfaces in the NP size larger than several nm.^51^

**Fig. 4:**
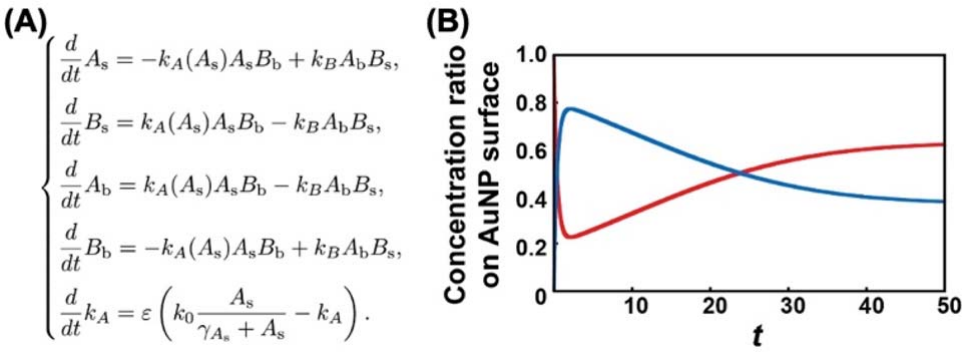
(A) The mathematical model of the ligand exchange reaction on gold nanoparticles. *A*_s_ and *B*_s_ are ligand A and B concentrations on the gold nanoparticle surface, respectively. *A*_b_ and *B*_b_ correspond to the bulk concentration of ligand A and ligand B. *k*_0_ is the maximum desorption rate of ligand A, *e* corresponds to the time constant, g_As_ is a positive constant. (B) The numerical result of the mathematical model (A) for initial data *A*_s_(0) = 1.0, *B*_s_(0) = 0.0, *A*_b_(0) = 0.0, *B*_b_(0) = 4.0, and *k*_A_(0) = 0.5. The red solid line shows the time evolution of the concentration ratio *A*_s_/(*A*_s_ + *B*_s_). The blue solid line shows the time evolution of the concentration ratio *B*_s_/(*A*_s_ + *B*_s_) where all parameters are *g*_As_ = 1.5, *e* = 0.1, *k*_0_ = 0.1, *k*_B_ = 0.5.

What causes this inversion of the changes in ligand content; that is, the unexpected increase in *T*_A_? Ligand exchange reactions include attachment and detachment. The equivalent attachment rate constant are expected from their similar chemical structures. Thus, the preferred replacement of C2- EG6-C11-SH with OH-EG6-C11-SH is supposed to result from a difference in detachment rate constant due to their thermodynamic stability. We have reported that free OH-EG6-C11-SH micelles show a clouding temperature of 59 °C, but OH-EG6-C11-SH attached on 5 nm AuNPs do not show thermo-responsive assembly formation up to 70 °C,^48^ suggesting the improved thermodynamic stability of alkanethiols attached on the surface. That paper also reports that increased hydrophobicity at the terminus leads to a lower *T*_A_ on AuNPs and clouding temperature as micelles. C2 (ethoxy)- and iC3 (isopropoxy)-terminated ligands showed a 31 and 18 °C difference between *T*_A_ on 5 nm AuNPs and clouding temperature as micelles, respectively. This finding supports the notion that a more significant stabilizing effect can be obtained *via* immobilizing OEG-ligands with a more hydrophilic terminus. This also indicates the competitive ligand exchange between two ligands with different stabilizing effects on the surface attachment causes the unfair surface composition. To support this idea, we construct a mathematical model and calculate the numerical simulation (Fig. 4A). Here, the detachment rate constant was set as *k*_A_ for ligand A and *k*_B_ for ligand B. In the model for Fig. 3A, ligands A and B are the OH-terminated and C2-terminated ligands, respectively. Somehow, only when *k*_A_ was set as a parameter depending on the surface ligand content of each molecule at the AuNP surface, we could obtain well-fitted curves of an overshooting-type as a potential mathematical model (Fig. 4B). Although the mechanism on this unfair ligand exchange remains uncertain, these results also support the importance of kinetic aspects, such as the detachment rate constant. In the same way, AuNPs@C1-EG6-C11-SH also showed a quick decrease in *T*_A_ (*i.e.*, corresponding to the ligand content at the AuNP surfaces) at 85 °C for 5 minutes in the presence of C2-EG6-C11-SH, followed by a small increase, indicating that there was a slight preference for C1-EG6-C11-SH to replace C2-EG6-C11-SH (Fig. 3B and S9†). The degree of the inverse increase in ligand content at the AuNP surfaces in this second stage of the reaction showed a good correlation with the difference in hydrophobicity at the ligand terminus. On the contrary, ligand content at the AuNP surfaces of AuNPs@C2-EG6-C11-SH in the presence of C1-EG6-C11-SH also quickly decreased for the first 5 minutes, and then continuously decreased to equilibrium without inverse changes. This result further supports the idea of a preferred ligand exchange from C2-EG6-C11-SH to C1-EG6-C11-SH (Fig. 3C and S10†). These kinetic analyses provide further support that the unfair ligand exchanges occurring in the competitive exchange reaction stage are related to their thermodynamic stability caused by the hydrophobicity of the ligand terminal, even when the differences are minor, such as hydroxy, methoxy, or ethoxy.

**Fig. 5:**
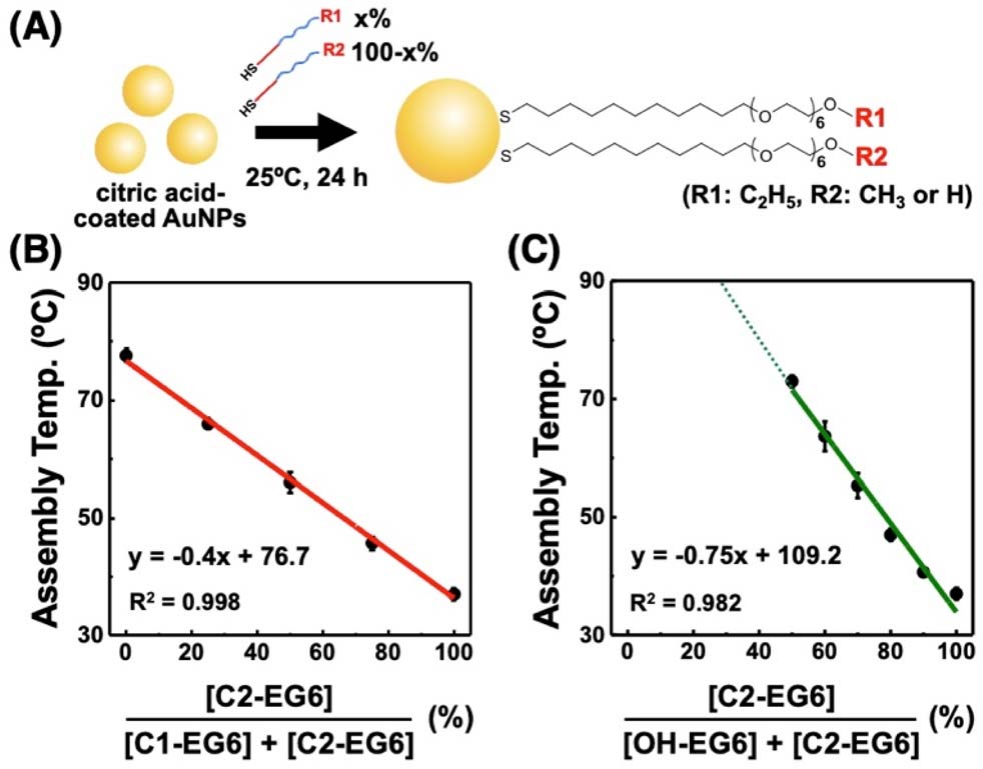
Controlled surface properties of the assembly temperature *via* fair surface modification of citric acid-coated AuNPs with alkanethiols. (A) Schematic illustration of this study. Assembly temperature of 10 nm AuNPs modified with a mixture of (B) C2-EG6-C11-SH and C1-EG6-C11-SH and (C) C2-EG6-C11-SH and OH-EG6-C11-SH. Error bars represent SD (*n* = 3). The *x*-axes in (B) and (C) represent the mixed ligand ratio used for the surface modification.

On heating at 55 °C, AuNPs@OH-EG6-C11-SH showed the decrease in ligand contents at the surface to be *ca.* 20% of the minimum produced by a 1 hour reaction in the presence of C2-EG6-C11-SH, followed by a slow increase (Fig. S11A†). The rate of change in surface ligand content was slower than that on heating at 85 °C. For heating at 25 °C, it took over 10 days to reach equilibrium, showing a temperature-dependent rate constant as expected (Fig. S11B†). AuNPs@C1-EG6-C11-SH in the presence of C2-EG6-C11-SH also showed similar changes in thermo-responsive assembly/disassembly at various heating temperatures, except for a relatively shorter time to reach equilibrium (Fig. S12†). This point could be related to the lower detachment constant of OH-EG6-C11-SH molecules with better thermodynamic stability. These results suggest that unfair ligand exchange between alkanethiols depends on thermodynamic regulation and is expected to be controlled by selecting appropriate reaction conditions, such as temperature and time. In other words, these are thought to be the key to fair surface modification.

### Surface modification with a minimal unfair ligand exchange

To realize fair surface modification with mixed ligands, we considered minimizing the unfair ligand exchange under appropriate reaction conditions. As a lower reaction temperature induces a slower ligand exchange and reduces the risk of competitive exchange between alkanethiols, we chose a reaction temperature of 25 °C. First, to confirm an adequate reaction time for full alkanethiol attachment by ligand exchange from citrate to alkanethiols, we mixed citrate-coated 10 nm AuNPs with C2-EG6-C11-SH and incubated them at 25 °C for 1, 3, and 24 hours. The results showed no significant change in *T*_A_ for all incubation times, suggesting that alkanethiol modification had finished within a few hours (Fig. S13†). Similarly, citrate-coated AuNPs were mixed with a mixture of C2-EG6-C11-SH and OH-EG6-C11-SH at the same concentration. AuNPs@(50% C2-EG6-C11-SH + 50% OH-EG6-C11-SH) also showed the same *T*_A_ of 70–71 °C for all incubation times (3, 6, and 24 hours), suggesting full ligand attachment for SAM and negligible unfair ligand exchange occurred in this time range (Fig. S14A†). Further incubation of these AuNPs over 20 days caused a significant increase in *T*_A_ due to unfair ligand exchanges (Fig. S14B†). This result supports the idea that unfair ligand exchange is negligible for at least several days at 25 °C. When AuNPs@(50% C2-EG6-C11-SH + 50% OH-EG6-C11-SH) prepared at 25 °C, having a *T*_A_ of 70 °C, were further incubated at 85 °C, their *T*_A_ was increased on incubation for 3 hours (Fig. S15†). These results strongly support our notion that moderate reaction conditions, such as several hours to one day at 25 °C, are suitable for surface modification.

To confirm fair surface modification with mixed ligands under moderate reaction conditions, citrate-coated 10 nm AuNPs were modified with a mixture of C2-EG6-C11-SH and C1-EG6-C11-SH at various mixing ratios at 25 °C for 24 h (Fig. 5A). These AuNPs showed apparent shifts in *T*_A_ between 36.5 °C for 100% C2-EG6C11-SH and 77.2 °C for 100% C1-EG6-C11-SH (Fig. 5B and Table S2†). This showed a good linear relationship, in which the correlation coefficient value of *R*^2^ is infinitely closer to 1, between *T*_A_ and the ligand mixing ratio (as the content of C2-EG6-C11-SH in this system), indicating that the actual composition of alkanethiols on the AuNP surface is in accordance with the ratios of the ligands applied for surface modification. Similarly, the *T*_A_ of C2-EG6-C11-SH mixed with OH-EG6-C11-SH also showed an excellent liner relationship, although the *T*_A_ was not totally determined due to the limitations in temperature range, when the OH-EG6-C11-SH content is over 50% (Fig. 5C). These results indicate fair surface modification with mixed ligands was performed *via* minimization of the unfair ligand exchange between alkanethiols.

Until now, surface modification of gold surfaces with alkanethiols has been widely performed and a long reaction time has been applied for the preparation of well-packed monolayers. The idea that a more prolonged reaction is better has been the prevailing view.^26^ Here, we showed the unfair ligand exchange between alkanethiols occurred over a long reaction time, even though alkanethiol SAMs possess a good stability. As this unfair exchange reduces the controllability or tunability of surface properties with mixed ligands, fair surface modification is desirable. Based on this fact, we have succeeded in perfect tuning of the thermo-responsive properties *via* well-controlled surface modification with mixed OEG-alkanethiols. As this study only investigated the combination of 10 nm AuNPs and OEG-alkanethiol derivatives and kinetic parameters are expected to change depending on the surface curvature and molecular packing on AuNPs, we need to tune appropriate reaction conditions for each sample. However, this insight regarding fair surface modification with mixed alkanethiols on gold nanoparticles through minimal unfair ligand exchange is expected to be widely applied to surface modifications with mixed ligands.

## Conclusions

In this study, we investigated the fair surface modification of the citrate-coated AuNPs with mixed OEG-alkanethiols. As this surface modification includes not only ligand exchange from citric acid to alkanethiols but also exchange between free alkanethiols and those immobilized on the AuNP surfaces, we clarified the kinetic aspects of both these exchange reactions based on their thermo-responsive properties. First, the latter reaction was investigated. Notably, ligand exchanges between alkanethiols occurred and slowly led to the unfair surface composition; *e.g.*, over a few hours at 85 °C or 20 days at 25 °C. On the contrary, the former ligand exchange from citric acid to alkanethiols was quickly completed within a few hours at 25 °C. This is a crucial insight for fair surface modification with mixed OEG-alkanethiols on the citrate-coated AuNPs *via* minimal unfair ligand exchange between alkanethiols under moderate reaction conditions, such as for ∼24 hour at 25 °C, despite the conventional idea that is a longer reaction time is better for well-packed SAM formation with alkanethiols on Au surfaces. Further, exchange reactions between alkanethiols allow us to reprogram the surface properties of alkanethiol-modified AuNPs. The insights obtained from this study can lead to not only precise tuning but also reprogramming of surface properties on gold surfaces with SAMs.

## Experimental section

### Materials

Citrate-protected AuNPs in aqueous solution (10 nm in diameter) were purchased from BBI Solutions (UK). OEG-Alkanethiol ligands having an oligo(ethylene glycol), alkyl tail, and thiol group with methyl and ethyl head (referred to as C1-EG6-C11-SH and C2-EG6-C11-SH, respectively), were purchased from ProChimia Surfaces, Sp. z o. o. (Poland). Alkanethiol ligands with a hydroxyl head, referred to as OH-EG6-C11-SH, and 4-(2-hydroxyethyl)-1-piperazineethanesulfonic acid (HEPES, buffer) were purchased from DOJINDO LABORATORIES (Japan). The OEG-alkanethiol derivative, OH-EG6-C12, was purchased from Tokyo Chemical Industry Co., Ltd. (Japan). Bis(p-sulfonate phenyl)phenylphosphine dihydrate dipotassium salt (BSPP) and thiol-terminated poly(*N*-isopropylacrylamide) with carboxylic acid (pNIPAM, Mn = 6000) were purchased from Sigma-Aldrich Co. LLC (USA). Tris(2-carboxyethyl) phosphine (TCEP-HCl, reducing agent) was purchased from Thermo Fisher Scientific Inc. (USA). All commercially available reagents were used without further purification.

### Surface modification of AuNPs with alkanethiols

The citrate-protected 10 nm AuNPs (9.4 nM) were concentrated up to 94 nM by centrifugation (20 000*g* for 45 min) and subsequent removal of the supernatant (900 μL). The concentrated AuNPs (100 μL) were mixed with the aqueous solution of the OEG-alkanethiol ligands such as C2-EG6-C11-SH with excess amounts (4 mM, 100 μL) containing TCEP as a reductant. After the addition of an aqueous solution of HEPES buffer (pH = 8.0, 10 mM) up to 1000 μL, the AuNPs were incubated for 24 hours at 25 °C. To remove the residual alkanethiol ligands and citrate, the modified AuNPs were washed 3 times by centrifugation (20 000*g* for 45 min), followed by the removal of the supernatant (900 μL) and the addition of HEPES buffer up to 1000 μL. The successfully modified AuNPs are referred as AuNPs@C2-EG6-C11-SH.

### Surface modification of AuNPs with pNIPAM

BSPP was added to the citrate-protected 10 nm AuNPs (9.4 nM) to a concentration of 5 mM and subsequently incubated at room temperature overnight with shaking. The incubated AuNPs (1000 μL) were mixed with an aqueous solution of the pNIPAM (4 mM, 100 μL) containing TCEP and then incubated for 3 hours at 85 °C. Then, the modified AuNPs were washed 3 times by centrifugation (20 000*g* for 45 min), followed by the removal of the supernatant (900 μL) and the addition of NaCl solution (20 mM) up to 1000 μL.

### UV-vis/NIR extinction spectrum measurement

The dispersed AuNP solution (3 mL, *ca.* 3 nM) was poured into a glass cuvette with a screw-cap to avoid solvent evaporation during measurements. UV-vis/NIR spectrum measurement were then performed with a spectrometer V-770 with PAC-743R automatic 6 position Peltier cell changer (JASCO Corp., Japan). The temperature-change measurements of the AuNP spectra were performed at each temperature (in 5 °C intervals) with changes at a rate of 1 °C min^−1^ followed by a waiting time of 5 minutes.

### Dynamic light scattering (DLS) measurement

The size distribution of the AuNPs was measured with a Zetasizer Nano ZS (Malvern Panalytical Ltd, UK). The emission wavelength for the measurement was 633 nm (4 mW He/Ne Laser). The temperature-change measurements for the AuNP sizes were performed at each temperature with a waiting time of 2 min. The assembly temperature was defined as the middle temperature between the temperature at which the size of the AuNPs showed a significant change and the highest temperature at which the AuNPs remained dispersed from a volume distribution as this helps to get an idea of the quantity of aggregation.

### Calculation of immobilized ligand concentration at the AuNP surface

The ligand density on the particle surface was set at 5–6 molecules nm^−2^.^48^ The surface area of a spherical gold nanoparticle can be calculated by *S* = 4π*R*^2^, where *R* is the radius of the sphere. From the calculated surface area, the number of ligands immobilized on a 10 nm AuNP was 1570–1880. From the AuNP concentration of 9.4 nM, the concentration of immobilized ligands was supposed to be 15–18 μM.

### Ligand exchange between alkanethiols on AuNPs

A free OH-EG6-C11-SH (1 mM, 10 μL) alkanethiol was added into the concentrated 10 nm AuNPs@C2-EG6-C11-SH (94 nM, 100 μL), followed by the addition of HEPES buffer up to 1000 μL. The extinction spectra of this mixture were measured by a UV-vis/NIR spectrometer on heating from 25 to 85 °C. Next, these AuNPs were washed 3 times by centrifugation (20 000*g* for 45 min), followed by spectrum measurement again. The size distribution on thermo-responsive assembly was measured by DLS. As a control, the OEG-alkanethiol derivative, OH-EG6-C12, without a thiol group was added into AuNPs@C2-EG6-C11-SH and measured by spectrometer and DLS. The peak wavelengths of spectra at every temperature of the above AuNPs were plotted for analysis of the effect of free ligands on their thermo-responsive temperature.

### Time-course experiment of the ligand exchange reaction

Kinetics of the ligand exchange were evaluated from a time-course experiment. Concentrated 10 nm AuNPs@OH-EG6-C11-SH (94 nM, 100 μL) was mixed with free C2-EG6-C11-SH (1 mM, 75 μL), followed by the addition of HEPES buffer up to 1000 μL. Then, these AuNPs were incubated at 85 °C for 3, 5, 30, 60, or 180 minutes. After incubation, AuNPs were quickly cooled down in an ice bath and purified at 4 °C *via* centrifugation three times (20 000*g* for 45 min). Assembly temperature (*T*_A_) was determined by DLS measurement. Then, the ligand content of at the AuNP surface was calculated based on the calibration curve, which is a linear relationship between the surface ligand content and *T*_A_, as shown in Fig. 4B.

## Data availability

All data generated or analyzed during this study are included in this published article and its ESI.†

## Author contributions

K. Xiong and H. Mitomo designed the research and experiments. K. Xiong performed experiments. M. Nagayama performed mathematical modelling and numerical simulation. The manuscript was written with contributions from all authors. All authors have approved the final version of the manuscript.

## Conflicts of interest

There are no conflicts to declare.

## Supplementary Material

NA-006-D4NA00270A-s001
