# Supplementary material for: Fair surface modification with mixed alkanethiols on gold nanoparticles through minimal unfair ligand exchange
Source: Nanoscale Adv. 2024 Jul 16;6(18):4583–90. doi: 10.1039/d4na00270a (PMC11385987; doi:10.1039/d4na00270a)
Supplement: NA-006-D4NA00270A-s001 [file NA-006-D4NA00270A-s001.pdf]

Electronic Supplementary Material (ESI) for Nanoscale Advances.
This journal is © The Royal Society of Chemistry 2024

Electronic Supplimentary Information

Fair Surface Modification with Mixed Alkanethiols on Gold Nanoparticles through Minimal Unfair Ligand Exchange

Kun Xiong,[a] Masaharu Nagayama,[b] Kuniharu Ijiro,[b] Hideyuki Mitomo[b]*

[a] Graduate School of Life Science, Hokkaido University, Sapporo 060-0810, Japan

[b]Research Institute for Electronic Science, Hokkaido University, Sapporo 001-0021, Japan.

E-mail: mitomo@es.hokudai.ac.jp

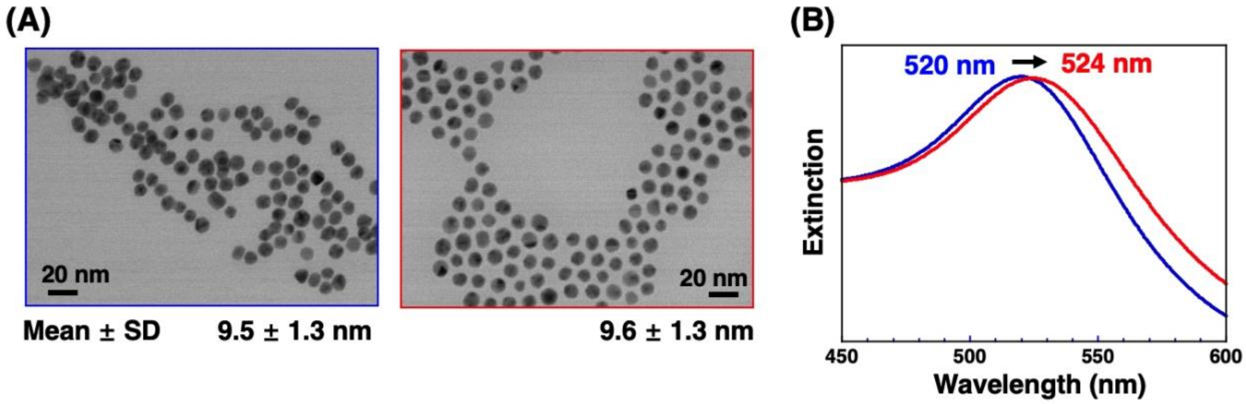

Figure S1. TEM images (A) and extinction spectra (B) of 10-nm AuNPs coated with citrate (blue) and OH-EG6 (red) as before and after reaction, respectively. The sizes in the TEM images indicate mean ± SD (*n* = 150 and 340). The numbers in the spectra indicate their peak wavelength.

Table S1. Hydrodynamic diameter and zeta-potential of AuNPs before and after surface modifications with C1-, C2-, and OH-EG6-C11-SH ligands.

Surface modifier	Zeta-Potential (mv)	Size*[1] (nm)
Citrate	-27.1	10.6
C1-EG6-C11-SH	-4.4	12.4
C2-EG6-C11-SH	-3.7	14.8
OH-EG6-C11-SH	-5.0	12.9

*[1] Size was determined as a main peak size in DLS size distribution by volume.

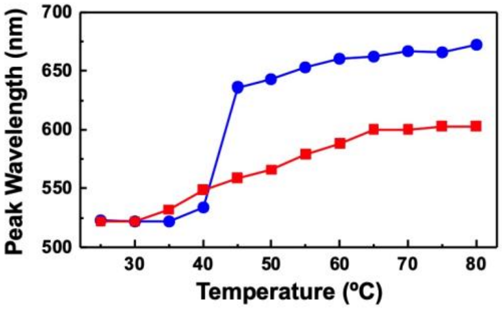

Figure S2. Extinction peak shifts of AuNPs@C2-EG6-C11-SH (blue) and AuNPs@pNIPAM-SH (red) upon heating. Thermo-responsive properties of AuNPs@pNIPAM-SH were measured in 20 mM NaCl aq.

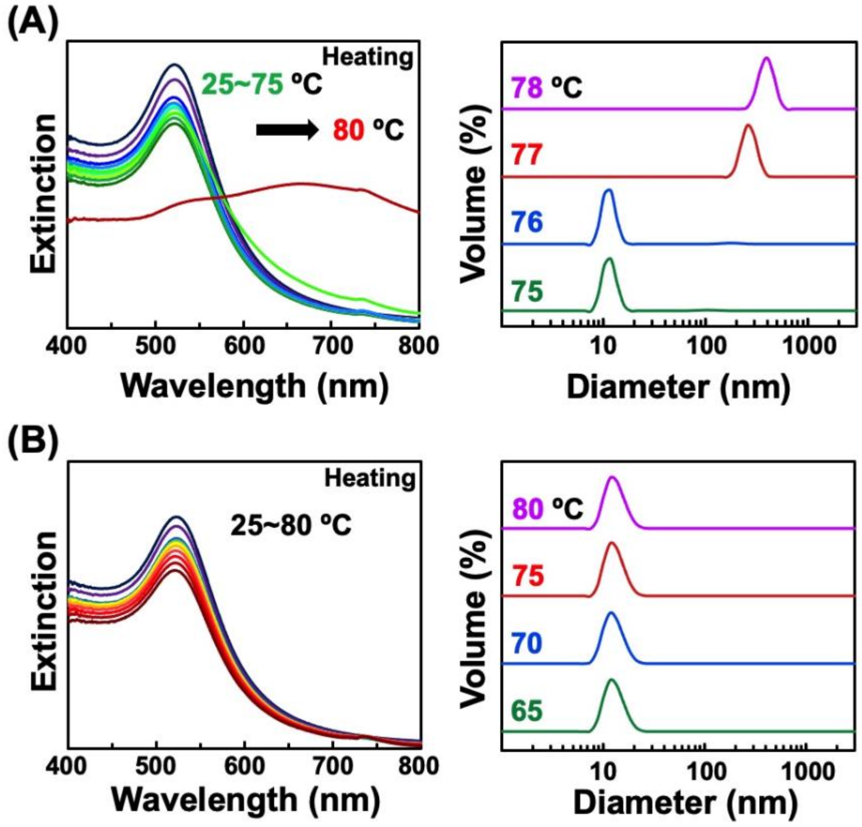

Figure S3. Thermo-responsive phenomena of AuNPs modified with (A) C1-EG6-C11-SH and (B) OH-EG6-C11-SH upon heating. Extinction spectra (left) and size distribution by DLS (right).

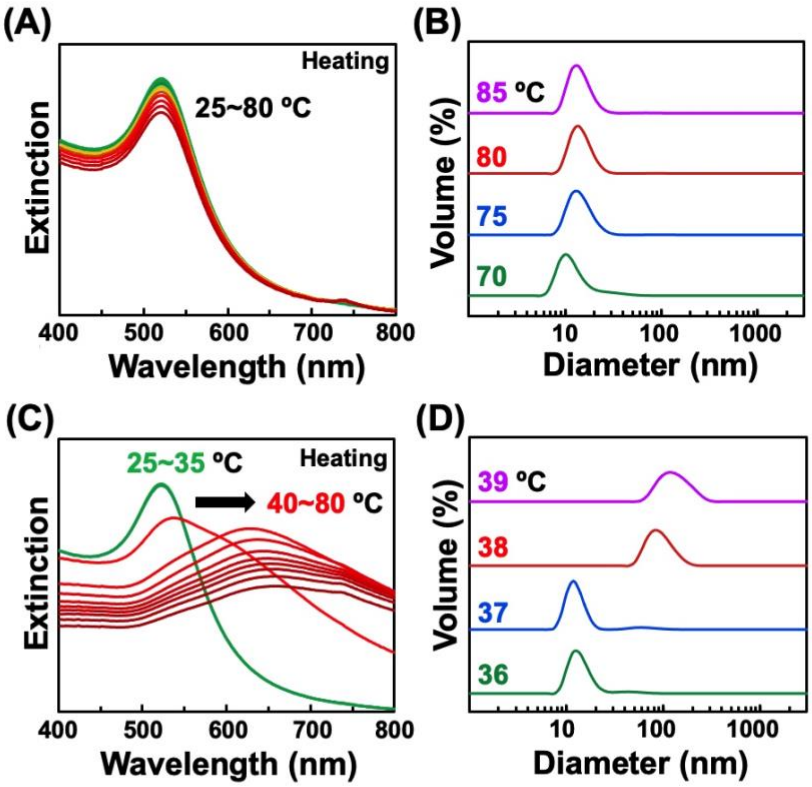

Figure S4. (A) Extinction spectra and (B) size distribution of AuNPs purified after measurements in Figure 2A in the absence of free alkane-thiols upon heating. (C) Extinction spectra and (D) Size distribution of AuNPs@C2-EG6-C11-SH upon heating in the presence of OH-EG6-C12.

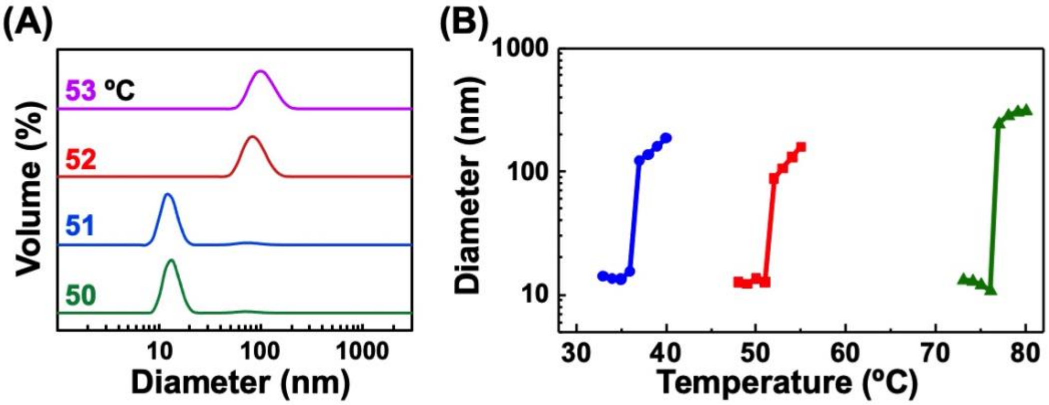

Figure S5. (A) Size distribution of C1-EG6-C11-SH-replaced AuNPs@C2-EG6-C11-SH determined by DLS and (B) comparison of thermo-responsive assembly temperatures of AuNPs@C2-EG6-C11-SH (blue), AuNPs@C2-EG6-C11-SH heated in the presence of C1-EG6-C11-SH at 85°C for 0.5 h (red), and AuNPs@C1-EG6-C11-SH (green).

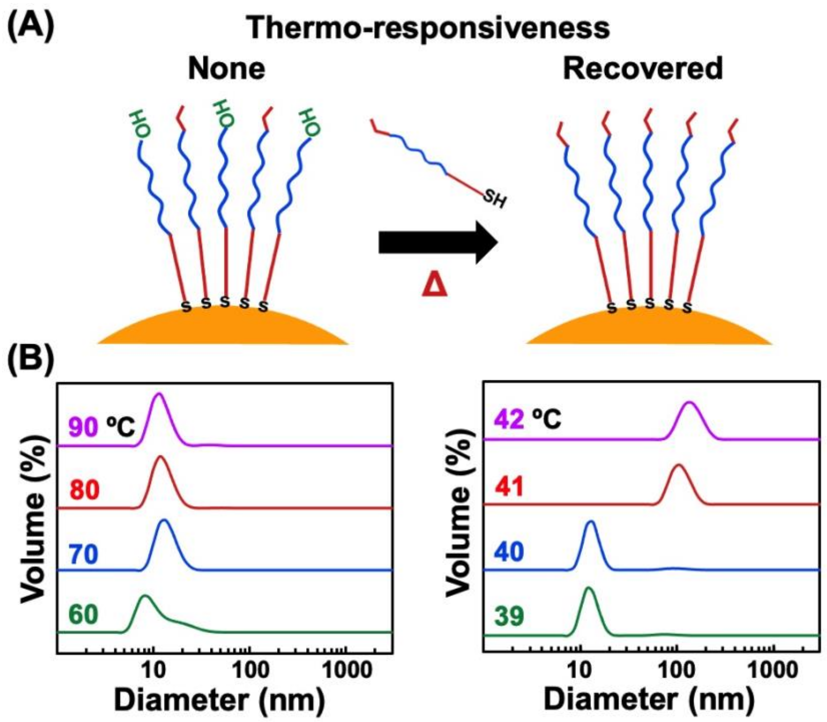

Figure S6. (A) Schematic illustration of the recovery of thermo-responsiveness by addition of C2-EG6-C11-SH as free ligands. (B) Size distribution of 10-nm AuNPs@C2-EG6-C11-SH reacted with OH-EG6-C11-SH after purification (left) and AuNPs further reacted with C2-EG6-C11-SH (right).

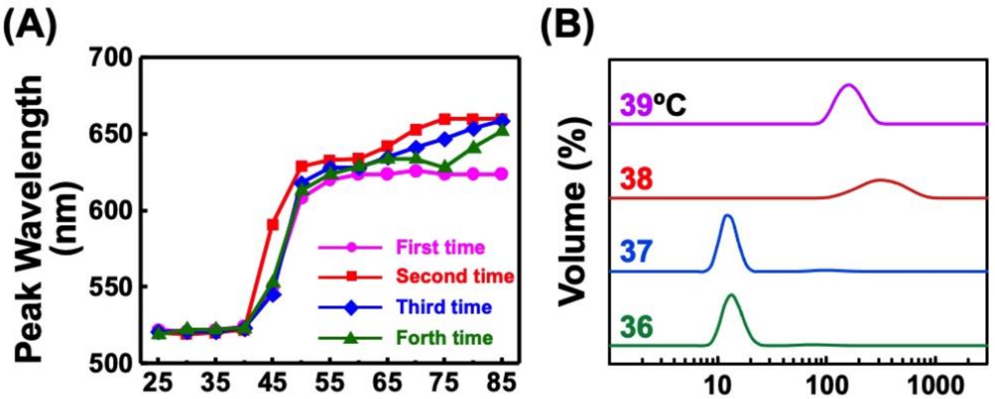

Figure S7. (A) Peak wavelength of 10-nm AuNPs@C2-EG6-C11-SH at each temperature over four cycles of heating. (B) Size distribution of the AuNPs after spectral measurement.

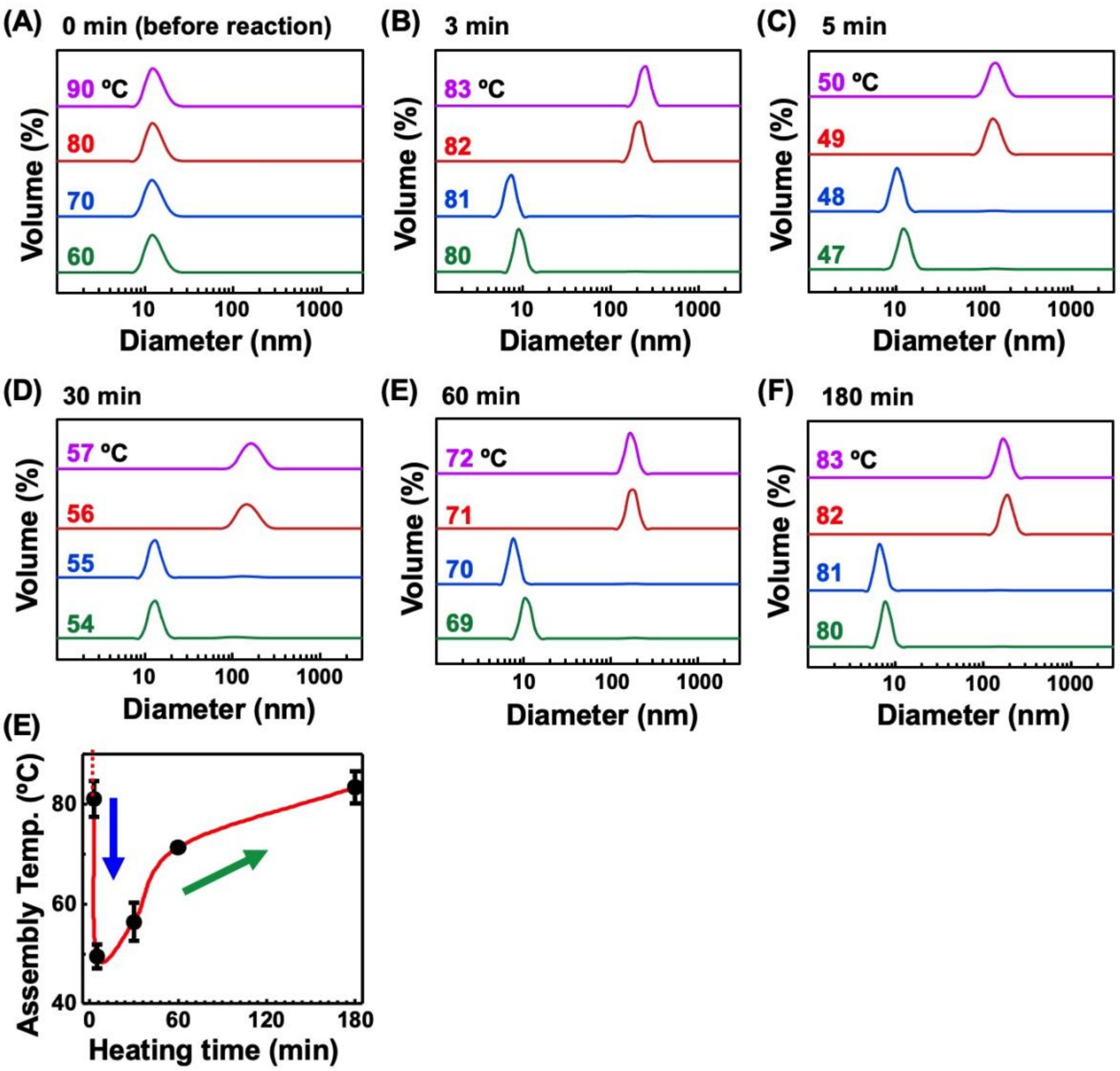

Figure S8. Reaction time-dependent changes in their thermo-responsive temperature of AuNPs@OH-EG6-C11-SH reacted with C2-EG6-C11-EG6 at 85°C. Size distribution at each reaction time: (A) before addition regarded as 0 minutes, (B) 3 min, (C) 5 min, (D) 30 min, (E) 60 min, and (F) 180 min. (E) Plot of assembly temperature at each time.

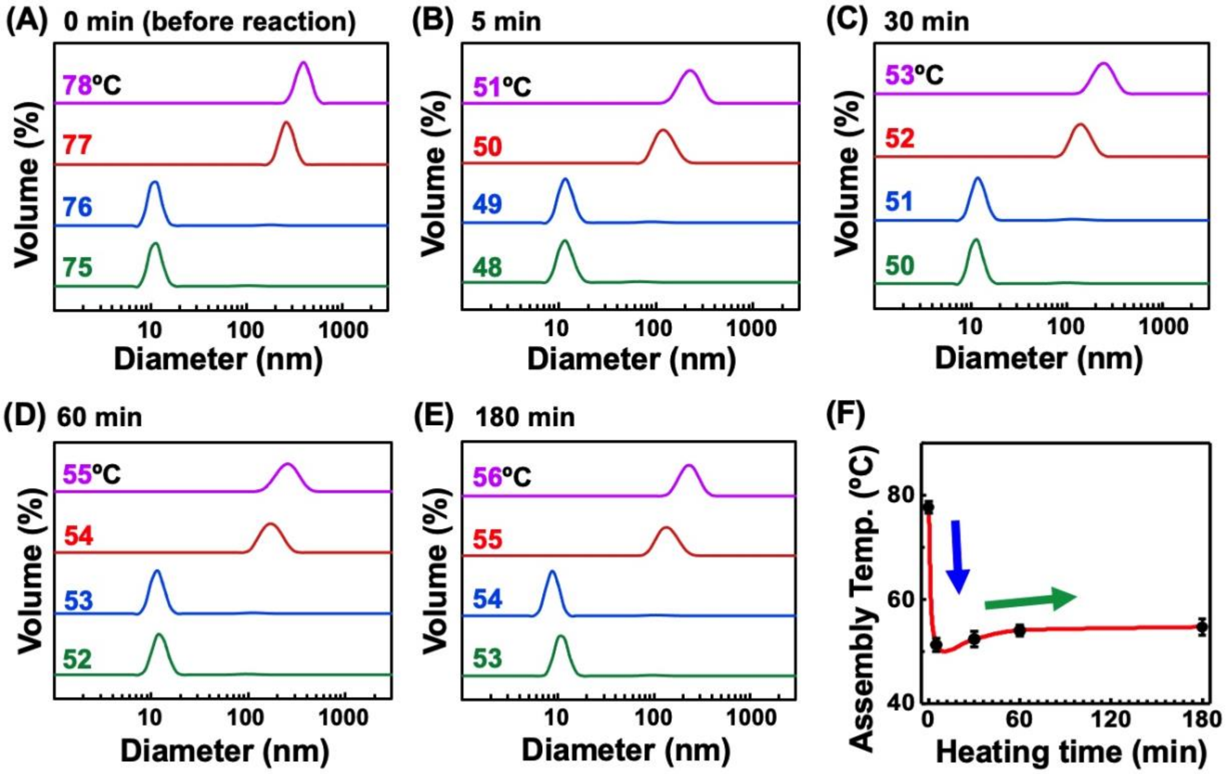

Figure S9. Reaction time-dependent changes in their thermo-responsive temperature of AuNPs@C1-EG6-C11-SH reacted with C2-EG6-C11-EG6 at 85°C. Size distribution at each reaction time: (A) before addition regarded as 0 minutes, (B) 5 min, (C) 30 min, (D) 60 min, and (E) 180 min. (F) Plot of assembly temperature at each time.

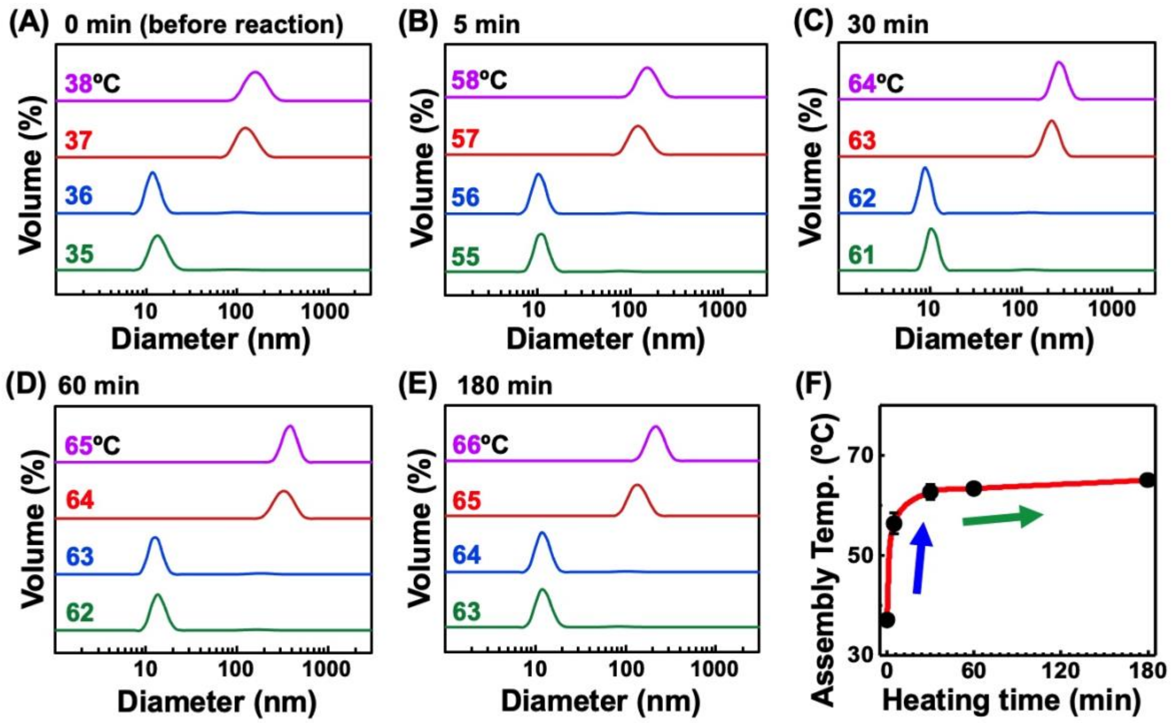

Figure S10. Reaction time-dependent changes in their thermo-responsive temperature of AuNPs@C2-EG6-C11-SH reacted with C1-EG6-C11-EG6 at 85°C. Size distribution at each reaction time: (A) before addition regarded as 0 minutes, (B) 5 min, (C) 30 min, (D) 60 min, and (E) 180 min. (F) Plot of assembly temperature at each time.

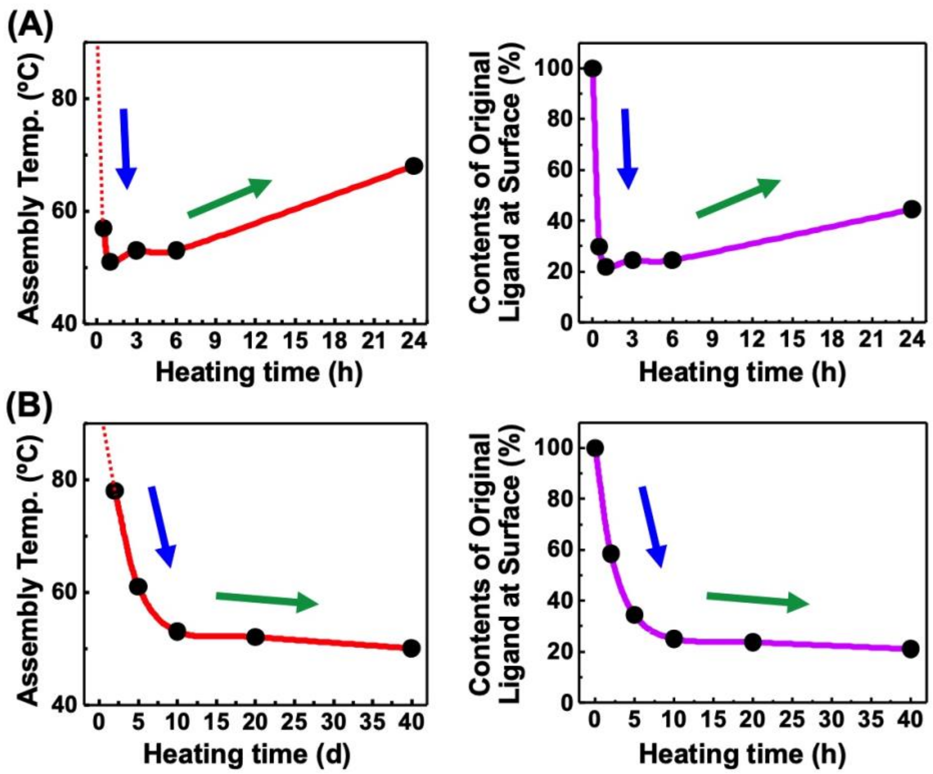

Figure S11. Assembly temperature changes (left) and ligand content changes at the AuNP surface (right) in the time-course experiment for10-nm AuNPs@OH-EG6-C11-SH reacted with C2-EG6-C11-SH at (A) 55°C and (B) 25°C.

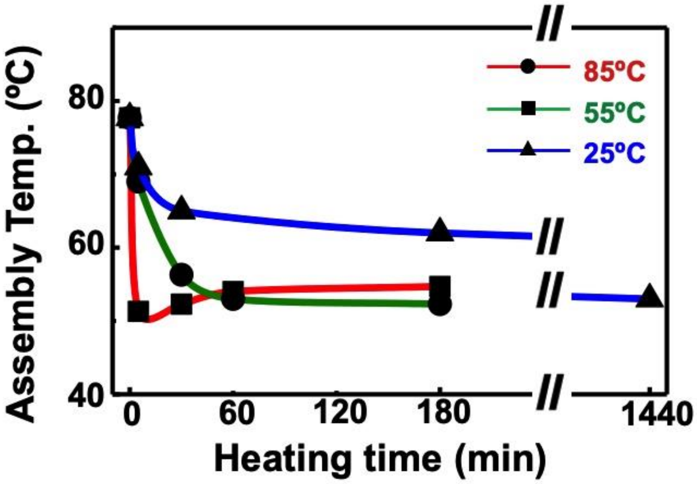

Figure S12. Assembly temperature in the time-course experiment for 10-nm AuNPs@C1-EG6-C11-SH by addition of C2-EG6-C11-SH at the ligand exchange temperature of 85°C, 55°C, and 25°C.

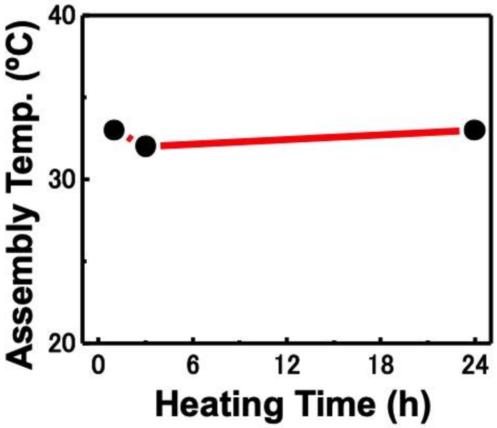

Figure S13. Thermo-responsive size distribution of (A) AuNPs@C2-EG6-C11-SH incubated for various times at 25ºC.

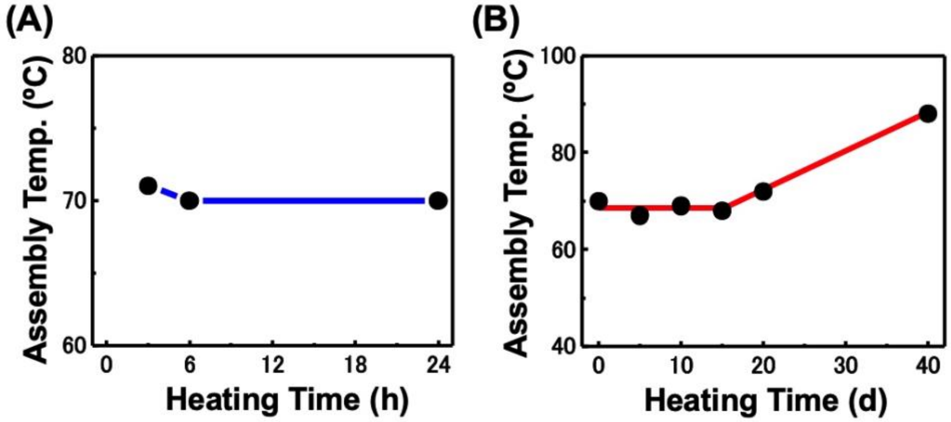

Figure S14. Assembly temperature changes in the time-course experiment for 10-nm AuNPs@ (50% OH-EG6-C11-SH + 50% C2-EG6-C11-SH) in the presence of a mixture of C2-EG6-C11-SH and OH-EG6-C11-SH at the same concentration at 25°C (A) within 24 hours and (B) over 24 hours.

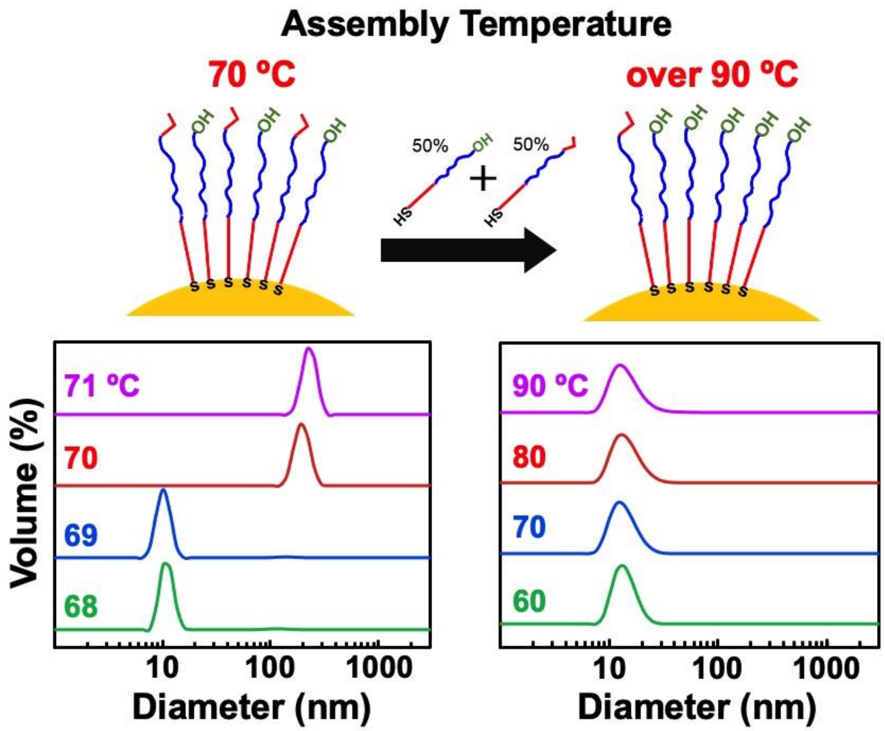

Figure S15. Assembly temperature change in 10-nm AuNPs modified with the mixed ligands of 50% C2-EG6-C11-SH + 50% OH-EG6-C11-SH upon heating at 85°C for 3 hours in the presence of the identical free ligands.

Table S2. Assembly temperature of 10-nm AuNPs modified with the mixed ligands at various ratios: (A) C1-EG6-C11-SH and C2-EG6-C11-SH and (B) OH-EG6-C11-SH and C2-EG6-C11-SH.

(A)

C2-EG6-C11-SH (%)	100	75	50	25	0
C1-EG6-C11-SH (%)	0	25	50	75	100
T_A (°C)[*1]	37.0 ± 1.0	45.7 ± 1.2	56.0 ± 1.7	66.0 ± 1.0	77.7 ± 1.2

(B)

C2-EG6-C11-SH (%)	90	80	70	60	50
OH-EG6-C11-SH (%)	10	20	30	40	50
T_A (°C)[*1]	40.7 ± 0.6	47.0 ± 1.0	55.3 ± 2.1	63.7 ± 2.5	74.8 ± 2.6

*[1] T_A was determined from three independent DLS analyses.
